# Artificial intelligence in interventional cardiology: a review of its role in diagnosis, decision-making, and procedural precision

**DOI:** 10.1097/MS9.0000000000003602

**Published:** 2025-07-18

**Authors:** Tochukwu R. Nzeako, Chukwuka Elendu, Gift Echefu, Olawale Olanisa, Adekunle Kiladejo, Emi Disrael Bob-Manuel

**Affiliations:** a Department of Cardiology, Christiana Care Hospital, Delaware; b Federal University Teaching Hospital, Owerri, Nigeria; c Department of Cardiology, University of Tennessee Health Science Center, Memphis, Tennessee; d Department of Internal Medicine, Trinity Health Grand Rapids, Michigan; e Division of Cardiology, Holyname Medical Center, Newark, New Jersey

**Keywords:** artificial intelligence, cardiology, cardiovascular diseases, clinical decision support, personalized care

## Abstract

Cardiovascular diseases significantly burden healthcare systems globally, necessitating innovative solutions to enhance diagnosis, treatment, and patient management. Artificial intelligence (AI) is no longer a distant promise in interventional cardiology but a rapidly emerging tool with growing clinical impact. AI-driven technologies can analyze vast amounts of clinical data, recognize intricate patterns, and generate clinically relevant, evidence-based recommendations, augmenting physician expertise and streamlining care. In diagnostics, AI enhances imaging interpretation and lesion assessment, while procedurally, it supports real-time guidance and catheter-based interventions. Its integration into decision support systems has improved risk stratification, early disease detection, and individualized treatment planning. AI also advances personalized medicine using predictive models to tailor interventions to patient-specific needs. Despite its promise, challenges such as costs, ethical issues, and the need for rigorous validation remain barriers to widespread adoption. Nevertheless, as AI advances, its integration into interventional cardiology is expected to transform care delivery, optimize outcomes, and improve system efficiency.

## Introduction and background

Cardiovascular diseases (CVDs) remain a leading cause of morbidity and mortality worldwide, with their prevalence continuing to rise over the past decade. This growing burden underscores the need for innovative strategies to enhance prevention, early diagnosis, and treatment to improve patient outcomes and reduce healthcare costs^[1]^. Effective CVD management requires a multifaceted approach, incorporating risk assessment, timely intervention, and continuous monitoring^[2–4]^. Technological advancements have significantly enhanced healthcare delivery in recent years, improving diagnostic capabilities, therapeutic precision, and long-term patient care^[5,6]^. However, with an aging population and an increasing incidence of cardiovascular conditions, there is an urgent demand for novel solutions to optimize clinical decision-making and procedural efficiency. Artificial intelligence (AI) has arrived as a notable force across multiple medical disciplines, including cardiology. AI refers to developing computer systems capable of performing tasks that typically require human intelligence, such as pattern recognition, problem-solving, and decision-making^[1,3,5]^.

To ensure conceptual clarity, we define key AI-related terms about interventional cardiology. Machine learning (ML) is a subset of AI that enables systems to learn patterns and improve performance from data without explicit programming. Within ML, deep learning utilizes multilayered neural networks to extract high-level features from large datasets and is especially impactful in image interpretation^[4]^. Based on current and historical data, predictive modeling uses statistical or ML techniques to estimate future outcomes, such as the likelihood of adverse cardiovascular events. AI-driven algorithms are an umbrella term that includes both traditional rule-based systems and modern learning-based models used for clinical decision support, image analysis, or procedural guidance. A key subset of AI, ML, enables systems to analyze vast datasets, recognize complex patterns, and make data-driven predictions without explicit programming^[7–9]^. Unlike traditional analytic tools, AI systems can learn from ongoing clinical feedback, allowing dynamic real-time patient data adaptation.

In interventional cardiology, AI enhances diagnostic workflows by automating the interpretation of angiograms, guiding catheter placement using real-time imaging feedback, and identifying high-risk coronary plaques using intravascular imaging modalities. ML algorithms, including convolutional neural networks (CNNs), have markedly improved the interpretation of cardiac imaging modalities such as echocardiography, cardiac magnetic resonance imaging (MRI), and computed tomography (CT) scans, facilitating faster and more accurate diagnoses^[10,11]^. Natural language processing (NLP) has also been integrated into clinical practice, automating medical documentation and enabling efficient analysis of electronic health records (EHRs). These advancements support physicians by extracting relevant clinical insights, identifying disease patterns, and optimizing patient management strategies^[12,13]^.

Additionally, robotics-assisted procedures have enhanced precision in interventional cardiology, minimizing human error and improving procedural outcomes. For example, robotic systems combined with AI can assist in precise stent deployment and reduce operator fatigue during complex procedures. AI-powered decision support systems analyze patient-specific data and provide tailored recommendations, allowing clinicians to make more informed treatment choices^[14,15]^. Beyond these applications, computer vision assists in interpreting cardiac imaging with greater accuracy, while cognitive computing enables AI-driven treatment planning and predictive analytics^[16,17]^. Affective computing, which focuses on recognizing and responding to patient emotions, is gaining relevance in personalized cardiology, helping to refine patient-centered care strategies.

**Figure 1.**
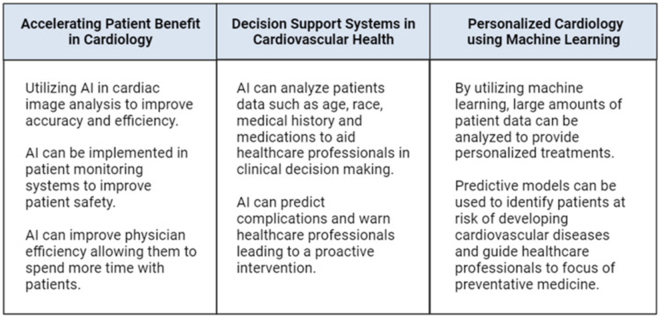
Summary of the uses of AI in the field of cardiology.

Furthermore, reinforcement learning – a branch of AI that improves decision-making through trial and error – can potentially optimize treatment protocols based on real-time data, leading to more adaptive and personalized interventions^[18]^. Recent clinical trials and pilot programs have shown how such AI approaches can reduce procedural times and improve post-procedure recovery, though large-scale validation is ongoing. AI’s contributions to interventional cardiology extend beyond diagnostics and decision support. Predictive modeling has demonstrated remarkable success in forecasting cardiovascular events, allowing for early intervention and risk stratification^[19,20]^. Moreover, AI-driven automation improves workflow efficiency, reducing physician workload and enhancing patient outcomes. However, despite its vast potential, integrating AI into cardiology faces critical challenges, including concerns over data quality, privacy, regulatory constraints, and ethical considerations. Addressing these hurdles is essential to ensure the responsible and effective implementation of AI-driven solutions in clinical practice^[21,22]^. Our review addresses AI’s role in interventional cardiology, distinguishing its contributions across diagnostic innovation, clinical decision-making, and intra-procedural assistance (Fig. 1 illustrates these domains and provides specific examples of how AI technologies are applied at each stage of patient care). The figure presents an integrated visual summary of AI’s capabilities, such as image-based diagnosis using CNNs, automated clinical decision support systems for individualized treatment planning, and robotics-assisted procedural navigation. We also discuss the challenges and future directions of AI integration, emphasizing its potential to reshape the landscape of cardiovascular care.

**HIGHLIGHTS**

- Artificial intelligence (AI) improves cardiac imaging, diagnosis, and treatment precision.
- Wearables with AI enable real-time, remote heart health monitoring.
- Data quality, bias, and ethics remain key challenges for AI in cardiology.

This figure illustrates the diverse applications of artificial intelligence (AI) across cardiology, including diagnostic interpretation, clinical decision support, risk stratification, imaging analysis, and procedural guidance. Notable AI-integrated tools include those that assist in evaluating global longitudinal strain (GLS) for myocardial function and predicting major adverse cardiovascular events (MACE). By leveraging machine learning algorithms and deep neural networks, AI enhances diagnostic precision, supports real-time clinical decisions, and facilitates personalized treatment strategies – especially in areas such as echocardiography, electrocardiogram analysis, and interventional cardiology planning – source: Authors’ creations.

## Data collection

**Figure 2.**
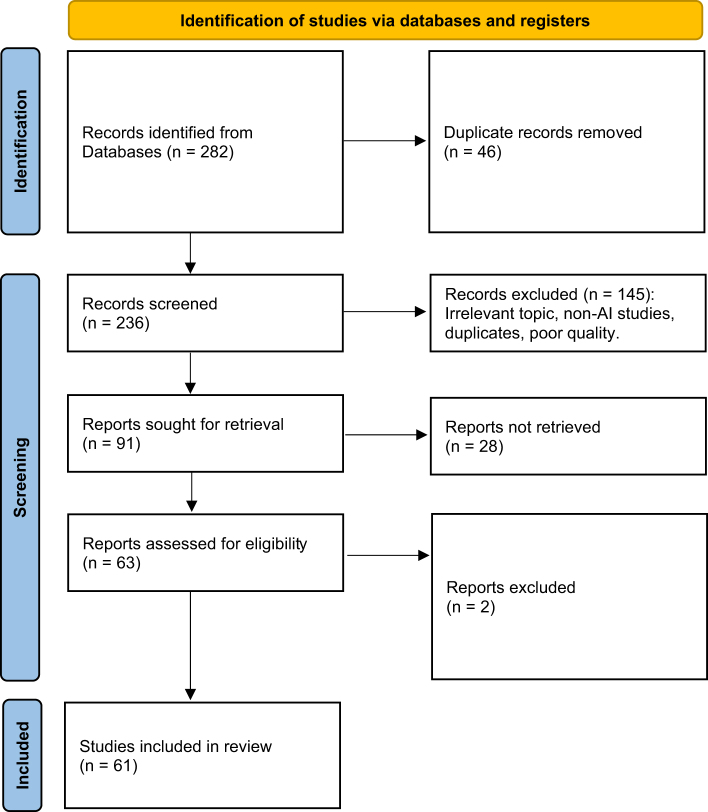
PRISMA flow diagram illustrating the process of study selection.

A structured literature review was conducted to identify relevant peer-reviewed research on integrating AI in interventional cardiology. Searches were performed using major academic databases – PubMed, SCOPUS, and Google Scholar – with clearly defined keywords such as “AI in cardiology,” “machine learning,” and “cardiovascular diseases” (Fig. 2). The scope was limited to publications from the past decade to reflect current trends and technological advancements.

The PRISMA flow diagram depicts the study selection process, outlining the identification, screening, eligibility assessment, and inclusion of studies considered in this review. Records were excluded for reasons including non-cardiology focus (*n* = 58), lack of AI relevance (*n* = 42), duplicate entries (*n* = 18), language/full-text issues (*n* = 17), and poor methodological quality (*n* = 10) – source: Authors’ creations.

Our review emphasized studies applying AI to interventional cardiology domains such as diagnostic imaging, clinical decision support, personalized treatment planning, and patient monitoring. Predefined inclusion criteria required that articles be published in English, peer-reviewed, and focused on applying advanced AI techniques – including ML or deep learning – in interventional cardiology settings. Exclusion criteria included non-peer-reviewed literature, purely theoretical models without clinical correlation, and studies unrelated to interventional cardiology. Eligible articles were required to incorporate advanced AI methods like ML or deep learning and to demonstrate practical implications in enhancing clinical workflows or outcomes.

Each selected study was reviewed for core methodologies, key findings, and clinical relevance. Quality assessment was based on factors such as study design (e.g. randomized trials, retrospective cohorts), sample size adequacy, transparency of AI modeling, and clarity of outcome measures. Studies with insufficient methodological detail or a high risk of bias were excluded.

Only methodologically sound, peer-reviewed publications were included. Rather than compiling data strictly quantitatively, a qualitative narrative synthesis was used to summarize key themes and identify trends, challenges, and future directions.

In line with current best practices for scientific integrity and the responsible integration of digital tools in research, we acknowledge the TITAN Guidelines 2025 for transparency in the reporting of AI, while noting that no generative AI tools were used in the preparation of our work^[23]^.

While efforts were made to ensure a thorough and balanced review, limitations were acknowledged. These include publication bias, heterogeneity among study designs, and the rapidly evolving nature of AI in healthcare, which may limit the inclusion of very recent but unpublished innovations.

## Accelerating patient care in cardiology through AI

AI integration in cardiology enhances patient care by improving diagnostic accuracy, streamlining decision-making, and optimizing procedural outcomes. AI-driven algorithms, particularly deep learning models such as CNNs, have demonstrated remarkable capabilities in cardiac image analysis^[24]^. These models have been successfully employed in interpreting cardiac MRI, CT scans, and echocardiograms, significantly improving diagnostic precision^[24]^. A study by Hannun *et al*^[25]^ highlighted the potential of AI in electrocardiogram (ECG) interpretation, where deep neural networks outperformed cardiologists in diagnosing ECG abnormalities and arrhythmias. The model achieved a high diagnostic performance, with an area under the receiver operating characteristic curve of 0.97 and an F1 score of 0.837, surpassing the average cardiologist’s score of 0.780. However, despite these impressive results, the study’s reliance on a single health system dataset raises concerns about potential dataset biases and limited generalizability. The lack of external validation across diverse populations further limits the applicability of these findings in broader clinical settings. These findings suggested that AI could enhance the accuracy of ECG analysis, reducing misdiagnoses and prioritizing critical cases for expert review^[25]^. Similarly, AI has been utilized to predict atrial fibrillation using wearable ECG monitors. By analyzing standard 10-second, 12-lead ECGs, AI algorithms identified electrocardiographic markers of atrial fibrillation even in normal sinus rhythm, achieving an area under the curve (AUC) of 0.87 and an overall accuracy of 79.4%^[26]^. Yet, this model’s performance may vary across different devices and population subgroups, and few studies have examined its long-term clinical utility. This innovation holds significant promise for early detection and management of atrial fibrillation, potentially improving patient outcomes.

Beyond ECG analysis, AI transforms echocardiography by minimizing human error and interobserver variability, particularly in assessing left ventricular systolic function and global longitudinal strain (GLS). AI-driven systems can accurately identify cardiac structures, calculate ventricular volume, and evaluate myocardial motion. In 2021, an AI-based model was developed to detect structural abnormalities in echocardiographic images, including valvular disorders^[27]^. Additionally, deep learning techniques have been employed to automate the calculation of left ventricular ejection fractions, improving the accuracy and consistency of cardiac function assessment^[28]^. While these studies show high diagnostic agreement with expert interpretations, they often lack large-scale clinical validation and real-world performance assessments, which are crucial before widespread implementation.

However, while AI has shown immense promise, challenges remain regarding its widespread clinical adoption, including algorithm validation across diverse populations, standardization of models, and seamless integration into existing clinical workflows. Future efforts should focus on refining AI models for broader applicability, enhancing interpretability, and fostering collaboration between AI systems and healthcare professionals to ensure reliable and actionable insights. AI’s potential extends beyond echocardiography to advanced imaging modalities such as cardiac MRI and CT scans. While AI applications in these areas are still emerging in cardiology, studies in other medical specialties have demonstrated its effectiveness. For instance, AI has been used to diagnose Alzheimer’s disease from MRI images^[29]^and detect lung cancer in CT scans with high accuracy^[30]^.

Nevertheless, direct extrapolation to cardiac applications is premature without thorough validation, as the complexity of cardiovascular structures and functional assessments poses unique challenges. These findings suggest that AI could be leveraged in cardiology to enhance the detection of cardiovascular pathologies in MRI and CT images (Table 1 overviews select AI applications in cardiac imaging, summarizing their diagnostic targets, reported accuracy, sensitivity, specificity, and areas requiring further development). By contextualizing these findings, the table highlights promising use cases – such as AI-assisted echocardiographic interpretation and plaque characterization on CT – and emphasizes key limitations and future considerations like model generalizability, integration into clinical workflows, and patient data protection^[31]^.

**Table 1:** Selected research on artificial intelligence applications in cardiology: use cases, outcomes, and implementation notes

Reference	AI technique employed	Clinical area	Reported outcomes	Remarks on clinical integration
[12]	Deep learning model for ECG interpretation	Arrhythmia detection via ECG	High classification accuracy (AUC: 0.97; F1: 0.837)	Emphasis on interpretability and clinical alignment
[14]	AI-assisted wearable ECG monitoring	Atrial fibrillation risk prediction	Sensitivity: 79%, Specificity: 79.5%, Accuracy: 79.4%	Need for validation across wider populations and settings
[21,32]	AI-driven imaging support in interventional planning	Coronary angiography and TAVR	Notable reductions in procedural time and complications	Algorithm robustness across procedural variations needed

This table highlights representative studies showcasing the role of AI tools in cardiologic diagnostics and interventions, with a focus on reported performance outcomes and translation into clinical workflows. Created by the authors based on synthesized findings from multiple studies^[12,14,23,105]^.

To further support this perspective, Table 2 provides a comparative summary of notable studies evaluating AI in interventional cardiology, outlining the AI modalities used, clinical applications, performance outcomes, and study limitations. These comparisons illustrate how AI-driven technologies are adapted to procedural contexts such as angiography interpretation, risk stratification, and catheter navigation. However, rigorous validation, customization for cardiovascular applications, and seamless clinical integration are required for successful implementation. The current body of literature often lacks randomized controlled trials or head-to-head comparisons with established diagnostic pathways, limiting the strength of the evidence. Future research should focus on tailoring AI models to cardiac imaging, ensuring their accuracy and reliability in real-world settings.

**Table 2:** Notable studies demonstrating artificial intelligence integration in interventional cardiology: tools, impacts, and challenges

Reference	AI technique	Clinical function	Reported benefits	Observed constraints
[33]	Deep convolutional networks	Coronary angiogram assessment	High diagnostic accuracy (>90%) for stenosis detection	Findings limited by single-center dataset
[34]	Gradient-boosted decision trees (XGBoost)	Pre-PCI risk evaluation	Improved prediction of major adverse cardiac events	Retrospective model; lacks prospective validation
[35]	NLP integrated with machine learning	TAVR procedural planning	Efficient EHR data extraction for risk estimation	NLP sensitivity to documentation style variations
[36]	Computer vision algorithms	Real-time catheter guidance	Decreased fluoroscopy exposure; enhanced procedural flow	Requires advanced imaging systems; may not be scalable
[32,37]	Reinforcement learning	Post-PCI pharmacotherapy adjustment	Adaptive antiplatelet strategy improved safety and adherence	Mostly tested in simulated environments

This table summarizes selected investigations highlighting how various AI modalities enhance clinical decision-making, risk stratification, and procedural execution in interventional cardiology, while noting current limitations in real-world implementation. Created by the authors based on synthesized findings from multiple studies^[100–104,105]^.

AI also transforms interventional cardiology by enabling more precise and personalized treatment strategies. ML models can analyze patient-specific data – including genetic factors, medical history, and previous therapeutic responses – to tailor interventions for conditions such as coronary artery disease (CAD) and heart failure^[38]^. A 2019 study emphasized the value of AI in predicting individual responses to antiplatelet therapy, such as clopidogrel, based on genetic variations, paving the way for more personalized cardiovascular treatment^[39]^. Similarly, AI-driven algorithms have been used to predict responses to statin therapy, leveraging ML to analyze genetic and clinical factors^[40]^. In catheterization laboratories, AI enhances procedural precision by guiding coronary angiography. AI-powered systems can optimize C-arm positioning based on patient-specific anatomical factors, reducing procedural time and radiation exposure^[31]^.

Furthermore, during transcatheter aortic valve replacement, AI algorithms assist in determining valve placement and sizing, minimizing complications and improving procedural efficiency^[31]^. AI has also been utilized in device placement for congenital heart defects, including atrial septal defects, ventricular septal defects, and patent ductus arteriosus closures, improving procedural success rates^[41]^. While these AI-driven solutions offer immense potential, their full integration into routine clinical practice requires further validation, refinement, and collaboration between AI developers and interventional cardiologists.

AI is revolutionizing chronic disease management by facilitating individualized treatment plans based on predictive analytics. By leveraging vast datasets, AI models can analyze patient-specific risk factors and optimize treatment strategies for heart failure and arrhythmias^[20,42]^. Moreover, in interventional cardiology, AI-driven decision-support systems enhance procedural planning, reducing complications and improving patient safety^[43]^. Beyond direct patient care, AI is streamlining administrative workflows, allowing clinicians to focus more on patient interactions. AI-powered systems assist in medical record documentation by automating data entry, coding, and error detection, improving accuracy and efficiency^[44]^. NLP tools enhance documentation by converting spoken notes into structured medical records, reducing clinician workload and minimizing administrative burden^[45]^. These advancements contribute to a more human-centered approach to medicine, enabling physicians to dedicate more time to direct patient care^[46]^. AI also plays a crucial role in patient monitoring by continuously analyzing real-time vital signs such as heart rate, blood pressure, and respiratory rate. AI-powered monitoring systems enable early detection of deteriorating conditions, allowing timely interventions and improved patient outcomes^[47]^. Hannun *et al*^[25]^ demonstrated AI’s ability to detect early signs of atrial fibrillation from ECG data, enabling proactive management of cardiac arrhythmias.

Furthermore, wearable devices with AI algorithms could track vital parameters remotely, offering continuous insights into patient health and facilitating early intervention for cardiovascular events^[48]^. A novel algorithm combining two event-related moving averages and fractional Fourier transform has been introduced for ECG signal analysis, demonstrating superior accuracy in detecting heart diseases compared to traditional methods. This study utilized a dataset encompassing over 10 000 patients, underscoring the real-world applicability of AI-driven diagnostic models^[49]^. AI has also been deployed to identify malignant arrhythmias, enhancing patient safety and clinical efficiency^[50]^. By rapidly analyzing vast amounts of patient data, AI reduces physician workload and improves diagnostic accuracy and treatment planning^[51,52]^.

## Decision support systems in cardiovascular health

Clinical decision support systems (CDSS) are essential tools in modern healthcare, designed to enhance patient care by delivering tailored clinical insights derived from individual patient data and broader health information^[53]^. Historically, clinical decision support systems (CDSS) have operated as computerized platforms that integrate patient-specific details with established clinical guidelines to generate evidence-based recommendations, ultimately supporting clinicians in making informed decisions^[54]^. Early iterations of CDSS, introduced alongside computer technologies in the 1970s, faced notable obstacles. Poor system integration and workflow disruptions led to limited adoption, as many clinicians found them cumbersome and time-consuming^[55]^. However, advancements in health informatics, particularly the incorporation of EHRs, computerized provider order entry, and sophisticated web applications, have significantly improved the functionality and usability of CDSS. Today, these systems are far more interactive and seamlessly integrated into daily clinical practice, contributing to streamlined workflows and enhanced patient outcomes^[56]^. AI has further propelled CDSS capabilities, particularly within medical imaging. AI-powered algorithms, including deep learning models and neural networks, now demonstrate exceptional proficiency in interpreting diagnostic images such as X-rays, CT scans, and MRIs, assisting healthcare providers with accurate and timely decision-making^[11,57]^. Beyond imaging, AI-enhanced CDSS can provide real-time, data-driven insights such as identifying potential drug interactions, optimizing treatment plans to minimize adverse effects, and predicting patient outcomes, all of which contribute to improving the quality of cardiovascular care. A notable study demonstrated that CDSS improved clinical practice in 68% of cases. Four key features were identified as independent predictors of success: automatic delivery of decision support within the clinician’s workflow, providing actionable recommendations alongside assessments, offering support at the point and time of decision-making, and employing computerized systems for execution. When all four factors were present, the systems achieved a 94% success rate in improving clinical practices^[58]^. Despite these advances, critical evaluation of AI-driven CDSS in medical imaging reveals opportunities and ongoing challenges. Although existing studies highlight substantial benefits, significant variability exists in the performance of different AI-CDSS models across various imaging modalities. This diversity underscores the need for further comparative research to assess these systems’ scalability, reliability, and generalizability in different clinical contexts. Addressing these gaps through future studies may help refine algorithms to improve transparency and interpretability, which are essential for clinician trust and widespread adoption. Strengthening these aspects could move AI-powered CDSS toward standardized and universally applicable solutions for imaging analysis and cardiovascular treatment planning. Beyond imaging, ML algorithms integrated into EHR systems are increasingly used to predict patient risks and outcomes, such as myocardial infarction (MI), sepsis, and related complications (Table 3)^[59,60]^. In preventive cardiology, AI-driven predictive analytics have demonstrated impressive accuracy by analyzing large datasets, including clinical records and diagnostic imaging, to assess risks and forecast cardiac events with greater precision than traditional methods^[61]^. For example, a large-scale study involving over 2.27 million patients, including 20 591 diagnosed with MI, compared deep learning and ML models against standard logistic regression using known risk factors. Although deep neural networks with random under-sampling yielded the highest classification performance, improvements over traditional methods were modest, with an F1 score of 0.092 and an AUC of 0.835. These findings suggest that, despite advanced algorithms, the predictive gains may be limited when relying on harmonized datasets, and overfitting remains a challenge due to the relatively low incidence of MI cases^[60]^. Similarly, the application of ML and deep learning in sepsis prediction through EHRs has shown promise. A review of 42 studies identified significant potential for early detection, highlighting the need for standardized data practices, consistent sepsis definitions, and improved model calibration^[59]^. These insights emphasize the need for further refinement of predictive models to support robust, scalable, and clinically meaningful cardiovascular health and preventive care applications. The growing integration of AI into CDSS has introduced various algorithmic approaches. Traditional rule-based CDSS has evolved to include ML techniques, such as supervised learning algorithms (e.g. support vector machines, random forests) that predict outcomes based on historical patient data and unsupervised methods (e.g. clustering) that identify novel patient subgroups and disease patterns^[62,63]^. In cardiovascular imaging, CNNs have demonstrated strong potential in improving diagnostic accuracy, particularly in analyzing complex imaging datasets. As AI-CDSS becomes more prevalent, the demand for explainable AI (XAI) systems has grown. XAI aims to demystify complex algorithms, offering clear insights into how decisions are made. Techniques such as decision trees and model-agnostic methods like LIME provide transparency and interpretability, helping clinicians understand and trust the recommendations generated by these systems^[64,65]^. This is crucial in high-stakes environments like cardiology, where clinical expertise must align with algorithmic outputs to ensure patient safety and optimal outcomes. While AI-guided CDSS offers significant advantages, their implementation is not without challenges. These systems improve decision quality, accelerate intervention times, and support personalized patient care through tailored recommendations^[66–69]^. However, their effectiveness highly depends on the quality and completeness of the data received. Inadequate or inconsistent data can compromise performance and lead to erroneous conclusions^[70]^. Additionally, deploying AI-CDSS requires considerable infrastructure, user training, and ongoing maintenance, which can be resource-intensive and costly^[71,72]^. Despite these hurdles, with thoughtful integration, investment, and continued research, AI-powered CDSS has the potential to reshape cardiovascular healthcare delivery by improving precision, efficiency, and patient outcomes.

**Table 3:** Clinical decision support systems (CDSS) leveraging artificial intelligence in cardiology: applications, effectiveness, and implementation challenges

Reference	AI-powered tool description	Clinical decision support role	Reported impact	Key implementation notes
[5]	Real-time CDSS using patient-specific data	Guiding care decisions at the bedside	68% enhancement in adherence to evidence-based care	Variability in model performance across settings
[16]	Predictive modeling for sepsis detection	Early identification of cardiac-linked sepsis risk	Promising early detection capabilities	Dependent on EHR data quality and interoperability
[32]	Prognostic tool for myocardial infarction	Anticipating MI events for preventive action	F1 Score: 0.092; AUC: 0.835 – modest gain vs traditional methods	Calibration affected by low event prevalence; model still discriminative

This table presents select studies evaluating AI-based CDSS in cardiovascular contexts, highlighting their clinical utility, quantitative performance, and limitations affecting real-world deployment. Created by the authors based on synthesized findings from multiple studies^[5,16,105]^.

## Predictive analytics for risk stratification and outcome prediction

Traditional risk assessment models in cardiology, such as the Society of Thoracic Surgeons risk score and the EuroSCORE, have been instrumental in predicting surgical outcomes. However, these models often rely on a limited set of variables and may not fully capture the complexity of individual patient profiles. For instance, a study highlighted that many predictive models overestimate risk, particularly in high-risk patients, suggesting limited utility in provider performance analysis and surgical decision-making^[73,74]^.

Integrating AI and ML into predictive analytics has significantly reshaped this landscape. AI algorithms can process vast datasets, uncovering patterns and correlations that may elude traditional analysis. A narrative review emphasized AI’s potential to improve disease progression, treatment response, and recovery predictions, thus enhancing patient outcomes^[19,20]^.

In interventional cardiology, AI-driven predictive models have shown promise across various procedures. For example, studies have applied ML algorithms to predict outcomes in patients undergoing percutaneous coronary intervention (PCI), TAVI, and those with CAD or cardiogenic shock – demonstrating improved accuracy over traditional risk scores, with AUCs ranging from 0.82 to 0.83^[21,22,24]^. Such models also support personalized treatment strategies and early interventions, especially in high-risk cases.

England’s National Health Service has embraced predictive analytics through tools like AI-ECG risk estimation (Aire), which leverages AI-based ECG interpretation – previously detailed in earlier sections – to detect subtle structural abnormalities. According to findings in The Lancet Digital Health^[25]^, Aire demonstrated 78% accuracy in predicting 10-year mortality.

Despite these advancements, challenges persist:

(a) Data quality and integration: The effectiveness of AI models depends on high-quality, comprehensive data. However, integrating data from multiple sources, such as EHRs, imaging, and wearables, remains difficult^[26]^.

(b) Interpretability: Many AI tools act as “black boxes,” making clinical decisions harder to validate. Enhancing model transparency is vital for clinician trust.

(c) Ethical and legal considerations: Issues around patient privacy, data security, and algorithmic bias demand robust regulatory frameworks^[27,28]^.

## Personalized cardiology empowered by ML

Personalized cardiology is an evolving field focused on tailoring medical treatments and interventions to meet the unique needs of individual patients. This patient-centric approach moves beyond generalized treatment protocols to strategies that account for genetic, clinical, and lifestyle factors. Notably, a 2015 study demonstrated how personalized genetic testing and risk assessment could uncover the complex pathophysiology underlying CVDs, enabling more targeted therapies and follow-up care^[75]^. Today, personalized approaches are being applied in managing conditions such as heart failure, hypertension, and MI, with a growing emphasis on integrating multiple patient-specific factors to optimize care^[76]^. Beyond conventional pharmacological and surgical interventions, personalized cardiology increasingly incorporates lifestyle modifications tailored to support long-term patient well-being^[77]^. ML has become a cornerstone of this precision-focused transformation in cardiology. By leveraging large datasets and advanced algorithms, ML has proven highly effective in identifying and predicting high-risk patients, such as those presenting with ST-elevation myocardial infarction, thereby enhancing risk stratification and enabling the design of individualized treatment plans^[78,79]^. In 2017, a deep learning model was developed with accuracy comparable to that of experienced cardiologists in detecting arrhythmias, facilitating timely interventions, and ongoing monitoring. This study also evaluated automated versus manual strain analyses in acute MI patients, finding that automated assessments of GLS and global circumferential strain closely matched manual measurements. Importantly, GLS emerged as an independent predictor of major adverse cardiac events, underscoring the promise of automation in improving diagnostic efficiency and clinical decision-making^[80]^. Beyond diagnostic support, ML has advanced remote monitoring and telemedicine by analyzing real-time data from wearable devices equipped with AI algorithms. These tools have successfully detected rhythm abnormalities like atrial fibrillation, allowing for proactive, personalized interventions. Furthermore, recent innovations have refined ECG-based algorithms for identifying sudden cardiac arrest. One novel approach combined CNNs with Boosting (BS) classifiers to achieve exceptional accuracy in detecting shockable rhythms, boasting an accuracy of 99.26%, sensitivity of 97.07%, and specificity of 99.44% – enhancing the performance of automated external defibrillators in emergencies^[26,81]^. While these developments showcase the promising potential of ML in cardiology, it remains critical to evaluate their real-world applicability. Despite impressive accuracy metrics in controlled studies, challenges persist in ensuring these algorithms perform reliably across diverse patient populations, healthcare settings, and clinical workflows. Potential biases in training data, scalability issues, and the practical impact on patient outcomes require careful consideration. Critical appraisals are essential to move from promising experimental results to meaningful clinical integration. In addition, ML techniques have contributed to personalized cardiovascular risk assessment through the automated analysis of cardiac CT scans and coronary artery calcium (Ca) scoring. These automated systems facilitate individualized cardiovascular risk profiling by accurately detecting coronary artery calcification^[82,83]^. One study utilized both calcium scoring CT and coronary CT angiography (CCTA) scans from 72 patients, finding that (semi)automatic methods accurately identified 52%–94% of calcification lesions, with positive predictive values between 65% and 96%. These findings highlight the feasibility of automated risk categorization, though challenges remain in detecting lesions at specific anatomical sites^[82]^. Another innovative approach employed supervised learning algorithms to quantify coronary artery calcification directly from CCTA scans, eliminating the need for a separate calcium scoring scan. Using convolutional neural networks (ConvNets), this method automatically localized the heart and quantified calcifications, achieving a sensitivity of 71% and just 0.48 false positives per scan. These results indicate strong agreement with reference annotations while offering the advantage of reduced radiation exposure^[83]^. However, as with other ML applications, variability in performance across patient subgroups and clinical settings warrants further validation. Future research should aim to refine these techniques across larger, more diverse cohorts and explore their seamless integration into routine clinical practice to optimize risk assessment and patient outcomes (Table 4). Predictive modeling is another pillar of personalized cardiology, offering tools to inform clinicians and patients about individualized health risks and guide decision-making. Traditional models such as the Framingham Risk Score have long been used to estimate the 10-year risk of CAD by incorporating variables like cholesterol levels, age, gender, smoking status, and blood pressure^[84–86]^. However, ML has allowed the development of more dynamic and nuanced predictive models, drawing on vast clinical and imaging datasets to refine risk predictions and enhance personalized care strategies^[17]^. For example, ML has been employed to predict all-cause mortality in patients suspected of CAD, providing actionable insights that empower clinicians to deliver targeted care based on individualized risk profiles^[87]^. These predictive tools signal a promising future for data-driven, patient-centered cardiology and are expected to drive significant improvements in healthcare quality over the coming decades^[88]^. Nevertheless, future efforts must prioritize validating these ML-driven models across broader patient populations to ensure their accuracy, fairness, and practical utility. Long-term studies are needed to measure the sustained impact of these technologies on patient care and health system performance. Interdisciplinary collaboration among clinicians, researchers, and data scientists will be critical to fully harnessing the power of predictive modeling in advancing personalized cardiology. Additionally, as ML becomes increasingly embedded in clinical decision-making, transparency through XAI will be vital. By clarifying how algorithms arrive at their conclusions, XAI enhances clinician confidence, supports informed patient discussions, and ensures that the insights driving personalized treatments are both interpretable and trustworthy^[89]^.

**Table 4:** Applications of machine learning in cardiology: use cases, predictive performance, and implementation considerations

Reference	Clinical focus	ML-based solution	Key findings	Translational challenges
[18]	STEMI prognosis in low-resource settings	Risk prediction model using Extra Trees algorithm	Sensitivity: 85%; AUC: 79.7%; accuracy: 75% – strong mortality stratification	Generalizability concerns and need to address data bias
[64]	Functional assessment via cardiac MRI	Automated strain analysis for GLS and GCS	GLS and GCS yielded strong predictive accuracy for MACE	Workflow delays due to post-processing; requires broader validation
[32]	Coronary artery calcium (CAC) quantification	Semi-automated CAC detection from cardiac CT	Detected 52%–94% of lesions; PPV: 65–96%; *κ* = 0.80–1.00 for risk classification	Missed distal lesions, false positives near ostia, ambiguities in image regions

The table summarizes machine learning strategies deployed in cardiology for risk prediction, cardiac imaging, and automated calcium scoring, with attention to predictive strength and clinical integration hurdles. Created by the authors based on synthesized findings from multiple studies^[18,61,105]^.

## AI-driven imaging and procedural guidance in interventional cardiology

In cardiovascular imaging, AI has been instrumental in automating tasks such as image interpretation and anomaly detection. Deep learning algorithms can analyze vast amounts of imaging data to identify patterns indicative of CVDs, thereby assisting clinicians in early diagnosis and treatment planning. For instance, AI-driven systems have been developed to assess cardiac function by analyzing echocardiographic images, providing quantitative measurements that aid in the evaluation of conditions like heart failure^[1–3]^. These systems reduce clinicians’ workload and minimize inter-observer variability, leading to more consistent and reliable assessments. AI enhances procedural guidance through real-time image analysis and decision support during interventional procedures. One notable application is in the dynamic road mapping of coronary interventions. Traditional methods rely heavily on the use of contrast agents to visualize coronary arteries, which can pose risks to patients with renal insufficiency^[4]^. AI-based approaches, such as deep learning models integrated with Bayesian filtering, enable tracking catheter tips in X-ray fluoroscopy without continuous contrast administration. This technique allows for creating dynamic coronary roadmaps that adjust to cardiac and respiratory motions, providing interventional cardiologists with accurate visual guidance while reducing the reliance on contrast agents. Another significant advancement is the development of AI-driven device tracking systems^[5–7]^. Accurate detection and tracking of interventional tools, such as guiding catheters, are crucial for procedural success. Challenges such as device obscuration, changes in the field of view, and patient movement necessitate robust tracking solutions. Self-supervised learning techniques have been employed to create models capable of understanding spatio-temporal semantics in interventional imaging. These models learn from vast datasets to predict device positions accurately, even under challenging conditions, thereby enhancing the safety and efficacy of procedures^[8]^. A common procedure in interventional cardiology, stent placement has improved through AI applications. AI-driven imaging allows for precise lesion length and vessel diameter measurement, aiding in selecting appropriately sized stents.

Furthermore, AI can predict the likelihood of stent restenosis by analyzing various patient and procedural factors, enabling personalized treatment strategies. During the procedure, AI assists in real-time monitoring of stent deployment, ensuring proper positioning and expansion, which are crucial for the long-term success of the intervention. The integration of AI into the cath lab extends beyond individual procedures. AI-driven data analytics enable the continuous monitoring of procedural outcomes and complications, fostering a learning environment where practices can be refined based on real-world evidence^[9–11]^. This continuous feedback loop contributes to the standardization of care and the reduction of variability in procedural outcomes. Moreover, AI-powered decision support systems assist clinicians in selecting the most appropriate interventions based on comprehensive patient data analyses, enhancing personalized medicine in interventional cardiology.

Furthermore, AI has been utilized in reconstructing interventional tools from limited X-ray projections, facilitating tomographic guidance during procedures^[12–14]^. Deep learning-based pipelines have been developed to extract interventional tools from minimal projections and reconstruct them in three dimensions. This capability provides clinicians with comprehensive spatial information about the tools relative to cardiac anatomy, improving navigation and precision during interventions. The integration of AI in interventional cardiology also extends to ultrasound navigation. Goal-conditioned reinforcement learning has been applied to guide ultrasound probes to desired anatomical targets, enhancing the acquisition of standard and interventional views^[15,16]^. This approach assists less experienced sonographers in obtaining high-quality images, thereby improving diagnostic accuracy and procedural planning.

### Current real-world applications of AI in interventional cardiology

While much of AI development remains in the research and pilot stages, several AI systems have already been integrated into real-world interventional cardiology workflows with promising results. One prominent example is HeartFlow’s FFR-CT, an FDA-approved tool that uses AI to calculate fractional flow reserve from CCTA images noninvasively. Clinical studies have demonstrated that HeartFlow FFR-CT improves diagnostic accuracy, reduces unnecessary invasive angiography, and guides appropriate revascularization decisions^[5,7,8]^.

Additionally, systems like Philips’ IntelliSpace Cardiovascular and Siemens’ AI-Rad Companion integrate AI algorithms into cath lab imaging suites, assisting clinicians in lesion quantification, automated contouring, and procedural planning. These platforms have been associated with reduced procedure times, enhanced imaging interpretation, and improved workflow efficiency^[9]^.

In Japan and parts of Europe, AI-assisted intravascular ultrasound (IVUS) and optical coherence tomography (OCT) interpretation tools are used in high-volume centers to optimize stent placement. Outcomes from these centers report improved stent expansion rates and fewer post-procedural complications^[10]^.

These examples underscore the fact that AI in interventional cardiology is not only theoretical but is already enhancing clinical practice in select settings. However, broader adoption depends on scalability, validation, and system-wide integration^[11]^.

Despite these advancements, challenges remain in the widespread adoption of AI in interventional cardiology. Issues such as data privacy, the need for large annotated datasets, and the interpretability of AI models are ongoing concerns. Moreover, integrating AI into clinical workflows requires rigorous validation and regulatory approvals to ensure safety and efficacy^[17,18]^.

## Quantitative performance of AI models in interventional cardiology

While many studies report promising results, synthesizing these findings clarifies their clinical utility and limitations. For instance, CNNs used for automated detection of CAD on angiographic images have reported impressive accuracy rates, with AUC values as high as 0.94 and diagnostic accuracy exceeding 90% in some cases^[1,2]^. Similarly, ML models applied to IVUS and OCT images have demonstrated sensitivity and specificity above 85%, often outperforming traditional assessment by interventional cardiologists^[3,4]^.

Predictive models for PCI outcomes, such as success prediction and restenosis risk, have shown moderate to high performance. Gradient boosting and random forest classifiers trained on clinical and procedural variables achieved AUCs ranging from 0.82 to 0.89 for predicting in-hospital adverse events^[5,6]^. Furthermore, ensemble learning methods combining patient history, imaging data, and hemodynamic parameters have attained up to 87% predictive accuracy, offering real-time decision support during procedures^[7]^.

In electrophysiology, AI algorithms analyzing electrocardiographic signals for arrhythmia detection and ablation planning have yielded accuracies between 88% and 95%, with some deep learning architectures reporting F1-scores greater than 0.90 in validation cohorts^[8,9]^. These quantitative indicators highlight the robustness of AI systems in supporting clinical workflows, especially in high-stakes environments such as catheterization labs.

Despite these promising outcomes, some variability exists across studies, particularly due to differences in datasets, validation strategies, and clinical endpoints. Moreover, external validation is often lacking, and performance in real-world settings remains underexplored in many models. Nonetheless, the consistent demonstration of high AUCs, sensitivity, and accuracy underscores the potential of AI to augment precision and efficiency in interventional cardiology^[10,11]^.

## Ethical considerations and data privacy in AI-integrated cardiology

Ethical considerations and data privacy are pivotal in AI-integrated cardiology, where the reliance on vast amounts of sensitive patient information – including EHRs, imaging data, and genetic profiles – raises substantial concerns about data security, patient confidentiality, and informed consent. The aggregation and processing of these large datasets heighten the risk of breaches, unauthorized access, and misuse^[29]^. Ensuring robust protection involves implementing advanced encryption methods, secure storage solutions, strict access controls, comprehensive cybersecurity protocols, and adherence to regulatory frameworks such as the General Data Protection Regulation. Equally important is transparent communication with patients regarding how their data is collected, stored, and utilized. This makes informed consent not merely a formality but an active process that respects patient autonomy^[30]^. This necessitates developing clear, accessible educational materials to help patients understand the scope of AI applications and associated risks, which can be complex and challenging to grasp without proper guidance.

Algorithmic bias presents another significant ethical challenge, as AI systems trained on datasets that lack diversity may fail to perform equitably across patient populations^[38]^. Such biases can perpetuate and even amplify existing healthcare disparities, leading to misdiagnoses or suboptimal treatment for underrepresented groups. This is especially concerning in interventional cardiology, where timely and precise decision-making is critical. Addressing this issue requires curating diverse and representative datasets, implementing bias detection mechanisms, and rigorous validation of AI models across various demographics^[39]^. Moreover, ensuring fairness in AI deployment also involves considering socioeconomic factors to avoid widening the healthcare gap between well-resourced and underserved populations, promoting equitable access to AI benefits globally. Patient privacy must also be safeguarded in AI applications requiring cross-institutional data sharing and real-time integration. Even with anonymization, re-identification risks persist, especially when large data sets contain complex variables^[5]^. This reinforces the need for continuous evaluation of privacy-preserving technologies and dynamic consent models that allow patients to control how their data is used over time.

Transparency and explainability in AI are essential for fostering trust among clinicians and patients. Many AI algorithms, particularly those using complex ML techniques, operate as “black boxes,” making it difficult to understand how they generate recommendations^[40]^. This opacity raises concerns about the reliability and accountability of AI-driven decisions, particularly when adverse outcomes occur. AI systems should prioritize explainable outputs and user-friendly interfaces to maintain ethical integrity, enabling clinicians to critically evaluate and trust the insights provided. This also supports preserving the patient-clinician relationship, ensuring that AI is a decision-support tool rather than a directive force, thereby upholding the nuanced judgment, empathy, and responsibility central to patient-centered care^[41]^.

The implications of AI-driven decision-making on physician accountability are another critical concern. As AI systems become more autonomous in generating diagnostic or therapeutic suggestions, ambiguities arise around liability when errors occur. Establishing clearly defined legal and ethical guidelines is essential to determine whether responsibility lies with the clinician, the institution, or the AI developer. This requires proactive policy development, including protocols for documenting AI contributions to clinical decisions and clear delineation of oversight roles^[15,17]^.

Accountability further complicates the ethical landscape of AI-integrated cardiology. As AI becomes more involved in clinical decision-making, it is essential to establish clear guidelines that delineate the responsibilities of healthcare providers, AI developers, and institutions, particularly in cases of diagnostic errors or adverse events. This involves creating monitoring frameworks for continuous oversight of AI performance and protocols for identifying and addressing errors^[42]^. Finally, the ethical use of AI requires ongoing education and training for healthcare professionals. Clinicians must have a technical understanding of AI systems and the ethical principles governing their use. This knowledge ensures they can appropriately integrate AI into practice while safeguarding patient welfare. Multidisciplinary collaboration among ethicists, clinicians, data scientists, and policymakers is essential to develop guidelines that align technological innovation with the core values of medical ethics, ensuring that AI in cardiology advances in a manner that is safe, equitable, and respectful of patient rights^[43,44]^.

## Integration of AI with robotics in interventional procedures

Interventional cardiology has traditionally relied on manual dexterity and real-time decision-making by clinicians during procedures such as angioplasties and stent placements. Robotic-assisted systems introduced a new paradigm, enhancing precision and stability during interventions^[45,46]^. Robotic systems can mitigate the limitations of human hands, such as tremors, and provide consistent performance during intricate procedures. For instance, robotic magnetic navigation systems utilize magnetic fields to steer catheters with high precision, reducing the physical strain on operators and potentially lowering radiation exposure. The incorporation of AI into these robotic systems further augments their capabilities. AI algorithms can analyze vast amounts of patient data, including imaging and physiological parameters, to assist in planning and executing procedures^[47,90]^. ML models, a subset of AI, can predict potential complications and suggest optimal intervention strategies based on historical data. This data-driven approach enhances decision-making, leading to personalized treatment plans tailored to individual patient profiles. One notable application of AI in robotic interventional cardiology is the autonomous navigation of endovascular interventions. Research has demonstrated that AI can assist in guiding catheters through complex vascular structures, potentially reducing procedure times and improving accuracy^[48,49]^. A systematic review highlighted the experimental use of AI for autonomous catheter navigation, indicating promising results in preclinical settings. Moreover, AI’s real-time ability to process and interpret medical imaging is pivotal in interventional procedures. When integrated with AI, advanced imaging technologies can enhance cardiac anatomy visualization, facilitating precise device placement and reducing the likelihood of procedural errors. For example, AI-enhanced imaging can assist in identifying optimal stent deployment sites by analyzing blood flow dynamics and vessel morphology^[50,51]^. Integrating AI with robotics also promises to reduce radiation exposure for patients and clinicians. Traditional fluoroscopy-guided interventions expose individuals to ionizing radiation, posing long-term health risks. Robotic systems, especially with AI, can optimize catheter paths and minimize the need for prolonged fluoroscopy, thereby reducing radiation doses. Despite these advancements, several challenges must be addressed to fully realize the potential of AI-integrated robotics in interventional cardiology.

Data privacy and security are paramount, as AI systems require access to extensive patient information to function effectively^[52,53]^. Ensuring that this data is protected against breaches is crucial. Developing robust algorithms that generalize across diverse patient populations remains a significant hurdle. Ethical considerations, such as the extent of autonomy granted to AI systems in clinical decision-making, also warrant careful deliberation. Furthermore, integrating AI and robotics necessitates comprehensive training programs for clinicians to operate these advanced systems effectively. Bridging the gap between technological innovation and clinical application requires interdisciplinary collaboration among engineers, computer scientists, and healthcare professionals. Continuous evaluation through clinical trials is essential to validate the safety and efficacy of these technologies before widespread adoption^[54,55]^.

## Challenges and future directions

Integrating AI into interventional cardiology holds immense potential but is accompanied by notable challenges. A central obstacle lies in obtaining diverse, high-quality datasets for AI development. Rare cardiac conditions and underrepresented patient populations pose significant data scarcity issues, limiting the generalizability of AI models^[5,91,92]^. Beyond data collection, safeguarding patient privacy remains critical. AI systems must be trained on appropriately anonymized datasets, and stringent security protocols are necessary to prevent breaches and unauthorized access. These privacy concerns are heightened by the potential for AI systems to inadvertently perpetuate biases present in their training data, which can compromise diagnostic accuracy and equity in patient care^[93,94]^. Ethical considerations further complicate AI adoption in cardiology. Patient data protection extends beyond collection, requiring robust encryption, controlled access, and ongoing surveillance to prevent misuse. Privacy-preserving techniques, such as federated learning, offer promising strategies to minimize data exposure while maintaining model performance.

Additionally, questions surrounding accountability and liability emerge when AI systems contribute to clinical decisions that may result in misdiagnosis or harm, raising complex medico-legal implications^[95,96]^. Financial and logistical barriers also challenge widespread AI implementation. High upfront costs for advanced hardware, software, and workforce training present significant burdens, particularly in resource-constrained healthcare settings^[97–99]^. Specific barriers include the need for substantial infrastructure investments – such as AI-enabled imaging platforms, reliable high-speed internet, robust data storage solutions – and continuous power supply and cybersecurity systems. Equally critical is the demand for comprehensive training programs to upskill clinicians, technicians, and IT personnel on using and maintaining AI technologies^[10]^. These training efforts must be sustained and tailored to local capabilities to ensure proper deployment and supervision of AI systems. In many African countries, including Nigeria, these challenges are compounded by limited digital infrastructure, inconsistent electricity supply, and low penetration of EHRs. Furthermore, the lack of local datasets hampers the development of context-specific AI tools. At the same time, reliance on models trained on data from high-income countries risks poor performance in African populations due to demographic and epidemiological differences^[12,13]^.

In addition to these obstacles, regulatory hurdles represent a significant barrier to the widespread adoption of AI in interventional cardiology. Many AI tools are classified as Software as a Medical Device, requiring stringent regulatory oversight before clinical use. In the United States, AI-based systems must meet U.S. FDA guidelines, which include evidence of safety, efficacy, and explainability^[14,15]^. However, dynamic or self-learning algorithms pose unique challenges for approval due to their evolving nature. Similarly, in the European Union, conformity with the Medical Device Regulation is necessary, and regulatory requirements continue to evolve to accommodate the complexities of AI. A lack of harmonized international standards often results in fragmented approval processes, which can delay implementation. Moreover, the absence of robust regulatory frameworks in many low- and middle-income countries impedes local deployment, with no clear pathways for assessing or approving AI technologies in healthcare^[16,17]^. Proactive policymaking, international collaboration, and adaptive regulatory models are needed to ensure that AI tools can be safely and efficiently brought into clinical practice without compromising patient safety.

Several solutions can be considered to overcome these barriers. Cloud-based AI platforms can mitigate infrastructure demands by outsourcing computational needs and simplifying maintenance requirements. Modular, scalable AI systems designed for phased integration can ease financial strain and allow gradual capacity building. Public-private partnerships should be leveraged to fund both infrastructure development and training initiatives. International collaboration and twinning programs can also help transfer knowledge and technical expertise to low-resource settings^[18,73]^. Beyond financial investment, AI integration necessitates restructuring existing workflows and infrastructure to ensure seamless interoperability with current systems. These transitions demand strategic planning, standardized protocols, and collaboration between clinicians, IT specialists, and policymakers to facilitate smooth and effective adoption. Another concern is the potential overdependence on AI in clinical decision-making. While AI excels at processing vast amounts of data, excessive reliance without proper human oversight could lead to diagnostic errors or oversight of contextual clinical nuances^[100,101]^. Maintaining a balanced, collaborative approach – where AI supports, rather than replaces, clinical judgment – is essential. Protocols for continuous validation of AI outputs, alongside mandatory human verification, are crucial to mitigate risks of overreliance. Cultivating a critical mindset among clinicians toward AI-generated insights will ensure that technology remains an adjunct, not a substitute, for medical expertise.

Furthermore, while AI demonstrates proficiency in interpreting structured clinical data, it struggles to account for external influences on patient health, such as social determinants and interpersonal dynamics. These complex, often unstructured factors are difficult to capture in conventional datasets, limiting the holistic understanding of patient well-being within AI models^[90,102,103]^. In low-resource settings, social and economic determinants – including access to care, geographic disparities, and health literacy – play an even greater role in patient outcomes. Future AI systems must be designed with the flexibility to incorporate such contextual variables to ensure equitable and effective decision support across diverse care environments. Future research must explore methods to integrate socio-environmental data into AI systems, improving personalized care in interventional cardiology^[46]^.

### AI in preventive cardiology and remote patient monitoring

AI is increasingly shaping the future of preventive cardiology and remote patient care by enabling early detection, risk mitigation, and continuous health monitoring beyond clinical settings. In preventive cardiology, AI algorithms can analyze vast amounts of patient data – ranging from EHRs and genetic profiles to lifestyle metrics – to identify individuals at high risk of developing CVDs^[28]^. These predictive models support the implementation of timely lifestyle interventions and personalized preventive strategies, thereby reducing the incidence and burden of heart disease. Additionally, AI-powered wearable devices and mobile health applications continuously monitor vital signs such as heart rate, blood pressure, and cardiac rhythm, alerting healthcare providers to early warning signs of deterioration^[29]^. In telemedicine, AI facilitates remote triaging, automated symptom assessment, and virtual consultations, expanding access to care for patients in underserved or remote areas. Integrating AI into remote patient monitoring platforms also enhances chronic disease management by promoting adherence to treatment plans, optimizing medication use, and enabling dynamic risk stratification based on real-time data^[30]^. These innovations underscore AI’s important role in transitioning cardiology from reactive to proactive care models.

### Overcoming financial and logistical barriers in low-resource settings

Innovative and scalable strategies are essential to strengthen AI adoption in cardiology across low-resource settings. Public-private partnerships can play a pivotal role in offsetting the high initial costs of AI infrastructure by pooling resources from governments, non-profits, and technology companies. These collaborations can facilitate the deployment of shared digital platforms, provide funding for training programs, and support localized data collection initiatives^[31]^. Moreover, open-source AI tools significantly lower financial barriers by offering cost-free alternatives to proprietary software, allowing customization for local needs without extensive licensing fees. Cloud-based AI solutions are also viable, reducing the need for on-site computational power and enabling remote updates and maintenance^[38]^. In addition, regional AI innovation hubs and pilot projects – tailored to specific cardiovascular health challenges – can serve as blueprints for sustainable implementation. These initiatives should be aligned with capacity-building programs that equip healthcare workers with the skills necessary to operate and supervise AI tools^[39]^ effectively. Ultimately, ensuring the success of AI in African cardiology requires not only technological adaptation but also strong governance structures and inclusive stakeholder engagement to bridge gaps in infrastructure, policy, and digital literacy.

Looking ahead, the future of AI in cardiology relies heavily on establishing clear ethical guidelines and robust security protocols for data usage. Comprehensive strategies – including encrypted storage, access controls, anonymization techniques, and transparent consent processes – will be fundamental to protecting patient privacy and maintaining public trust in AI-driven healthcare^[104]^. Regulatory oversight must keep pace with technological advancements. Rigorous validation, transparency in AI decision processes, and accountability mechanisms are essential to ensuring safety and efficacy. In Africa, the absence of regionally unified regulatory frameworks for AI in healthcare presents an additional hurdle. Collaborative efforts between governments, healthcare institutions, and academic bodies will be critical to establishing localized standards that support safe AI deployment^[48]^. Proactive regulation will be key to mitigating potential harms while maximizing the benefits of AI innovations in cardiology.

Finally, continuous education and training for healthcare professionals are imperative. As AI systems evolve, clinicians must remain informed and competent in utilizing these tools responsibly. Ongoing professional development will empower practitioners to interpret AI outputs accurately, integrate them seamlessly into patient care, and uphold the ethical standards of modern medicine^[105]^. With thoughtful navigation of these challenges, AI is poised to transform interventional cardiology, enhancing diagnostic precision, optimizing decision-making, and improving procedural outcomes while safeguarding the core values of patient-centered care.

## Concluding remarks

AI rapidly transforms interventional cardiology, offering innovative solutions that enhance patient care across multiple domains. From advanced cardiac imaging and personalized treatment planning to real-time monitoring and decision support, AI-driven technologies contribute to more accurate diagnoses, optimized therapies, and improved patient outcomes. As these tools continue to evolve, they hold significant promise in reshaping the prevention, management, and monitoring of CVDs on a global scale. However, successfully integrating AI into clinical practice depends on overcoming key challenges, including ensuring data quality, protecting patient privacy, and addressing ethical and regulatory concerns. Robust oversight, transparency, and security protocols are essential to foster trust and safeguard patient welfare. With careful implementation and ongoing collaboration among clinicians, researchers, and policymakers, AI has the potential to reduce the global burden of cardiovascular disease and elevate the standard of cardiac care, ultimately improving health outcomes for patients worldwide.

## Data Availability

This published article and its supplementary information files include all data generated or analyzed during this study.
